# Structural, optical and electronic properties of Ni_1−*x*_Co_*x*_O in the complete composition range

**DOI:** 10.1039/d0ra09128f

**Published:** 2020-12-09

**Authors:** Kiran Baraik, Ashok Bhakar, V. Srihari, Indranil Bhaumik, C. Mukherjee, Mukul Gupta, A. K. Yadav, Pragya Tiwari, D. M. Phase, S. N. Jha, S. D. Singh, Tapas Ganguli

**Affiliations:** Synchrotrons Utilisation Section, RRCAT Indore 452013 India kiranb@rrcat.gov.in; High Pressure and Synchrotron Radiation Physics Division, BARC India; Laser and Functional Materials Division, RRCAT Indore 452013 India; Laser Technology Division, RRCAT Indore 452013 India; UGC-DAE CSR Indore 452001 India; Atomic and Molecular Physics Division, BARC India; Homi Bhabha National Institute Anushakti Nagar Mumbai 400094 India

## Abstract

Crystallographic and electronic structures of phase pure ternary solid solutions of Ni_1−*x*_Co_*x*_O (*x* = 0 to 1) have been studied using XRD, EXAFS and XAS measurements. The lattice parameter of the cubic rock-salt (RS) Ni_1−*x*_Co_*x*_O solid solutions increases linearly with increasing Co content and follows Vegard's law, in the complete composition range. A linear increase in the bond lengths (Ni/Co–O, Ni–Ni and Ni–Co) with “*x*”, closely following the bond lengths determined from virtual crystal approximation (VCA), is observed, which implies that there is only a minimal local distortion of the lattice in the mixed crystal. The optical gap of the ternary solid solution determined from diffuse reflectivity measurements shows neither a linear variation with Co composition nor bowing, as observed in many ternary semiconductors. This trend in the variation of optical gaps is explained by probing the conduction band using XAS at the O K-edge. We have observed that the variation in the onset energy of the conduction band edge with “*x*” is very similar to the variation in the optical gap with “*x*”, thus clearly indicating the dominant role played by the conduction band position in determining the optical gap. The variation in the intensities of the pre-edge peak in the XANES spectra measured at Ni and Co K-edges, and the L_1/2_ peak in XAS spectra measured at Ni and Co L-edges, is found to depend on the unoccupied O 2p-metal-(Ni/Co) 3d hybridized states and the bond lengths.

## Abstract 2

The optical gap of Ni_1−*x*_Co_*x*_O solid solutions neither varies linearly with Co composition nor shows any bowing in the complete composition range. The nature of this variation of the gap is governed by the position of conduction band edge.

## Graphical abstract

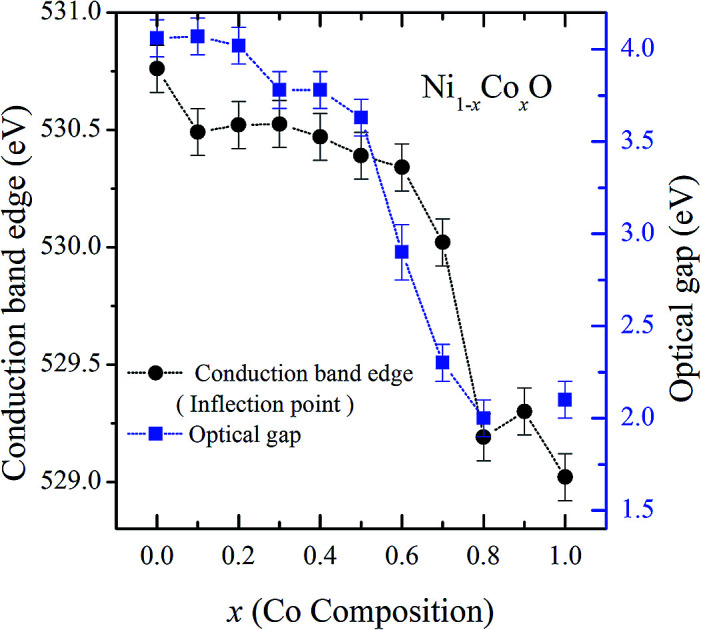

## Introduction

Transition metal oxides (TMOs) exhibit a wide range of properties, most of which originate from the strong 3d electron–electron correlation in the transition metals. These properties are not only interesting from a fundamental point of view, but also have been behind several applications. A few of them include giant magneto resistance (GMR), high *T*_c_ superconductors, metal to insulator transition, p-type transparent semiconductors and systems for anti-ferromagnetic spintronics.^1,2^ In the large class of TMOs, the bivalent oxides, NiO and CoO, and their ternaries, have applications in photodetection, resistive switching, energy storage in batteries, transparent electrodes, electrochromic smart windows, anti-ferromagnetic spintronics and solar cells.^2–5^ Pure NiO and CoO belong to the group of type-II anti-ferromagnetic (AF2) materials with a magnetic moment of 1.9 *μ*_B_ on the Ni site and 3.8 *μ*_B_ on the Co site, respectively.^6,7^ The NiO has a cubic (*a* = 4.173 Å) rock-salt (RS) crystal structure (*Fm*3̄*m*) above the Néel temperature (*T*_N_ = 523 K). Below *T*_N_, due to the magnetostriction effect, the structure undergoes a weak cubic-to-rhombohedral (*R*3̄*m*) distortion with the contraction of cubic unit cell along the [111] axis. The cubic (*a* = 4.261 Å) RS structure of CoO under goes to a monoclinic (*C*2/*m*) (*a* = 5.18190(6) Å, *b* = 3.01761(3) Å, *c* = 3.01860(3) Å) crystallographic distortion below the Néel temperature (291 K).^7^

NiO and CoO form a homogeneous solid solution in the cubic RS crystal structure, when annealed at temperatures above 780 °C. In the intermediate temperature range of 600–700 °C, co-existence of mixture of NiO–CoO and Ni_3_O_4_–Co_3_O_4_ solid solution has been observed. At calcination temperatures below 500 °C, only solid solution of nickel cobaltite NiCo_2_O_4_ with spinel structure has been reported.^8,9^ Recently, a formation process for 2D nanosheets of nickel cobalt oxide system having single phase cubic RS structure has been studied by *in situ* liquid phase transmission electron microscopy, which can have applications in sensing, catalysis and other surface enhanced applications.^10^ A superlattice consisting of alternating ultra-thin layers of NiO and CoO is found to be in a cubic RS structure for the thickness below 5 nm for each layer, and the lattice parameter is found to be in between NiO and CoO lattice parameter values.^11^ Further, the superlattice exhibits an intermediate value of Néel temperature in between the *T*_N_ values of CoO and NiO, because of strong interlayer exchange interaction.^12^

The one electron band theory which does not include the exchange and electron correlation effects, is insufficient to describe the electronic structure of TMOs. The band theory often predicts, no band gap, or gaps which is an order of magnitude smaller than the gaps observed experimentally. Density functional theory (DFT) with generalized gradient approximation (GGA) for exchange and correlation terms, predict a band gap of 0.3 eV for NiO, and predict CoO to be metallic.^13^ The experimentally observed insulating nature of the TMOs was explained by Mott and Hubbard theory. According to this theory, if the exchange and d–d Coulomb interaction energy, involving charge fluctuation of the type d_i_^*n*^d_j_^*n*^ → d_i_^*n*−1^d_j_^*n*+1^ (where i and j denote two TM sites), is larger than the one electron dispersion band width, then a correlation gap of the order of *U* (*U* = d_i_^*n*−1^ + d_j_^*n*+1^ − 2d_i_^*n*^d_j_^*n*^) is formed, with the Fermi level within this gap. This theory predicts an insulating gap or correlation gap of 10 ± 3 eV for NiO, and this is referred to as d–d transition Mott-insulator type.^14^ However, most of the theoretical calculations (such as, cluster model with configuration interaction,^15^ SIC-LSD^16^ and constrained LDA^17^), predict the gap of 3.5–6 eV for NiO and 2.5 to 7 eV for CoO. This gap has been attributed to the transfer of charge from ligand O 2p to metal (Ni, Co) 3d, resulting in a charge transfer gap (*Δ*). The charge transfer nature of the gap for these divalent oxides has been confirmed by different experiments, and the magnitude of the same has also been determined. Experimentally determined values of the charge transfer gap for NiO and CoO are found within the theoretically determined range of values mentioned above. The values of correlation gap (*U*) have also been determined experimentally and theoretically, and are found to be 7–10.5 eV for NiO and 4.7–11 eV for CoO.^1,14–18^ From the values of charge transfer gap and correlation gap obtained from the theoretical and experimental analysis, it is now established that the NiO belongs to the class of charge transfer insulator. However, even though, mostly CoO has been considered a charge transfer insulator, the nature of its insulating gap is still not clear, because of the scattered values of insulating gap. It is generally assumed that CoO belongs to a mixed class of Mott-insulator and charge transfer insulator.^1^

Although there have been studies on NiO and CoO that have been mixed with other non-magnetic oxides like MgO^19^ and spinel structured nickel cobaltite (NiCo_2_O_4_), but no report exists on a detailed study of single phase NiO–CoO system. The wide difference in the values of *T*_N_ between these two divalent oxides and the large band gap of both NiO and CoO, make this mixed system a potential candidate for the application in transparent anti-ferromagnetic spintronics with tunable band gap and Néel temperature.^20^

The main aim of the present work is to analyze the crystal and local structure, electronic structure and the optical properties of the Ni_1−*x*_Co_*x*_O system in the complete composition range. The structural analysis of phase pure Ni_1−*x*_Co_*x*_O solid solutions has been carried out using X-ray diffraction (XRD), X-ray absorption near edge structure (XANES) and Extended X-ray absorption fine structure (EXAFS). The lattice parameter linearly increases with Co incorporation in NiO following the Vegard's law. Bond length of near neighbor (NN) and next near neighbor (NNN) determined using EXAFS, are found to closely follow the values determined from XRD data under the Virtual crystal approximation (VCA). The variation of Néel temperature and the optical gap with composition have been determined by Differential scanning calorimetry (DSC) and Diffuse reflectivity spectroscopy (DRS) measurements, respectively. Even though a linear variation of structural parameters and Néel temperature are observed with composition variation, throughout the complete composition range, the variation of the optical gap deviates significantly from a linear behavior. This non-linear variation of the gap is explained in terms of the variation of the conduction band edge with composition, which has been determined by absorption measurement at O K-edge. For the completeness of the analysis of conduction band with XAS, study of pre-edge peak in XANES spectra measured at both the Ni and Co K-edges and the core level XAS at L-edges of Ni and Co have been presented.

## Experimental details

**Fig. 1:**
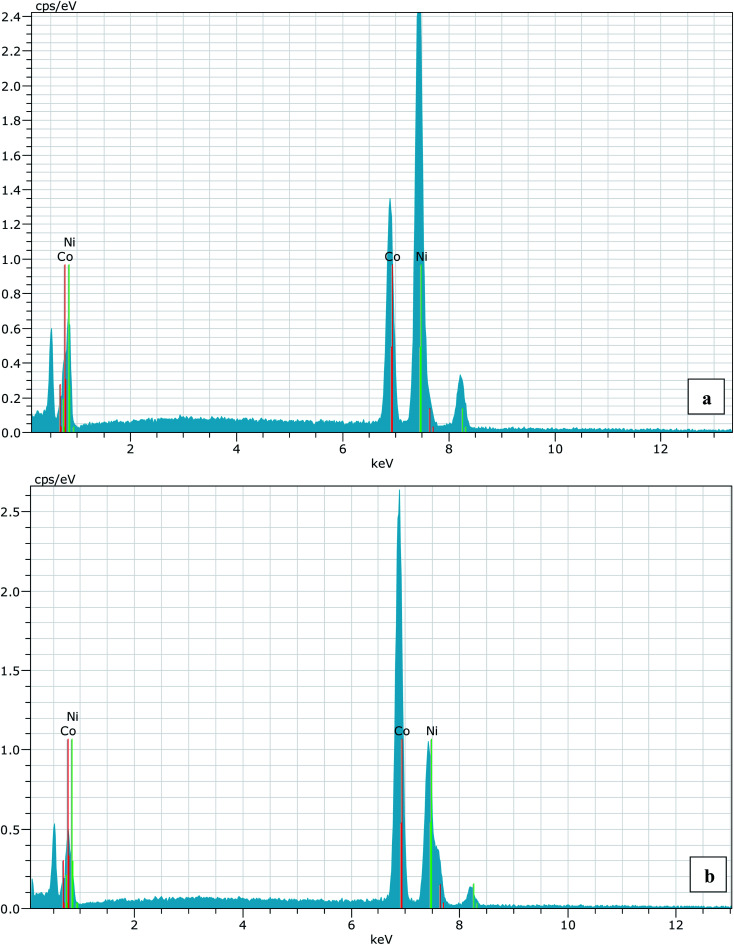
(a) EDS spectrum for 30% (nominal value) Co substitution in NiO and (b) is for 70% (nominal value) Co substituted NiO sample. The average Co composition in both the cases are found to 30.23% and 68.44%, respectively.

A series of Ni_1−*x*_Co_*x*_O (*x* = 0, 0.10, 0.20, 0.30, 0.39, 0.50, 0.59, 0.68, 0.78, 0.87 and 1.0) ternary solid solutions with varying Co composition has been synthesized using solid state reaction method. Formation of phase pure solid solution from the mixture of NiO and CoO powder was expected because of their similar crystal structure (FCC) and the similar values of ionic radii (Ni^2+^: 0.69 Å and Co^2+^: 0.72 Å), when annealed at temperature higher than 800 °C.^8,9^ The solid solution samples were prepared by mixing high purity NiO (99.998%) and CoO (99.995%) powders homogeneously in their molar ratio and pellets of 20 mm diameter were made by applying a force of ∼8 tons. The pellets were then annealed for ∼6 hours at 1300 °C before quenching them in liquid nitrogen. A secondary phase of Co_3_O_4_ along with Ni_1−*x*_Co_*x*_O solid solution, for *x* beyond 0.5, is observed when the pellets were cooled slowly from 1300 °C to room temperature.^6^ The Co content in the solid solution has been determined from Energy Dispersive X-ray spectroscopy (EDS) using Philips XL30CP having Bruker detector with Xflash. The EDS spectrum of two samples with the nominal composition of Co: 30% and 70%, are shown in Fig. (1). The crystalline structure, lattice parameters and the phase purity of the ternary solid solution samples were determined by XRD measurement at extreme conditions X-ray diffraction (ECXRD) beamline (BL-11), Indus-2, synchrotron radiation source (SRS), using a beam of wavelength of ∼0.5 Å.^21^ The lattice parameters of the samples were determined with LeBail fitting using Fullprof software.^22^ EXAFS measurements were been carried out to get the information about the local structure of Ni_1−*x*_Co_*x*_O crystals. These measurements were performed, at Ni and Co K-edge, at the scanning EXAFS beamline (BL-09), Indus-2, under ambient condition.^23^ The variation in the bond length of the ternary solid solution with Co incorporation in NiO have been evaluated. The behavior of the conduction band of Co substituted NiO bulk samples has been studied with soft X-ray absorption spectroscopy (SXAS) by recording the spectra at oxygen K-edge, Ni and Co 2p edge (L_3/2_ and L_1/2_ edges) at soft X-ray absorption beamline (BL-01), Indus-2.^24^ Total electron yield (TEY) has been chosen as a mode of SXAS data collection at the ambient temperature with energy resolution better than 300 meV. The Néel temperature, *T*_N_, of the solid solutions has been estimated using DSC (SETARAM DSC92).^25^ The discontinuity in the thermograms obtained from the DSC measurement of the solid solutions, gave the crystallographic and magnetic transition temperature for the samples. The DSC set-up used for the measurement can follow the phase transformation involved in the range of room temperature to 670 K. The optical gap variation in the series of Ni_1−*x*_Co_*x*_O bulk samples has been determined by DRS (CARY 5000 spectrophotometer using diffuse reflectance accessories (DRA)). Kubelka–Munk equation was used to determine the optical gap of the samples from the DRS spectrum.

## Results and discussion

**Fig. 2:**
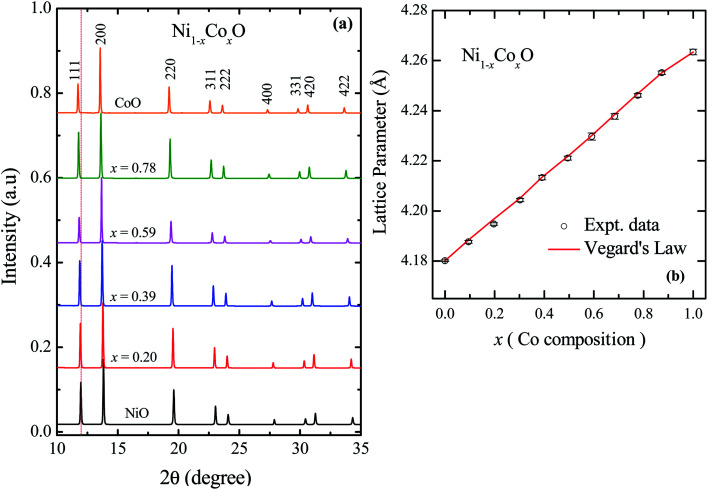
(a) XRD data from Ni_1−*x*_Co_*x*_O ternary solid solutions. The Bragg's peaks have been identified to be related to cubic RS crystal structure in whole composition range of Co. (b) Variation of lattice parameter with Co composition for ternary solid solutions, Ni_1−*x*_Co_*x*_O. The error bar is within the symbol.

The XRD patterns of Ni_1−*x*_Co_*x*_O solid solutions, recorded at room temperature, for different values of “*x*” is shown in Fig. 2(a). The lattice parameter obtained for NiO from the LeBail fitting of the XRD data was 4.1801(3) Å, which matches very well with data of JCPDF card no. 471049, and other reports.^26,27^ Rietveld refinement of the XRD data confirms the *Fm*3̄*m* space group of NiO. All the diffraction patterns obtained for increasing Co composition, could also be fitted with the *Fm*3̄*m* space group (cubic RS crystal structure). No additional phase could be detected in the XRD patterns in any of the samples, confirming the presence of phase pure cubic RS crystal structure for all the compositions. XRD data shows a monotonic decrease in the 2*θ* values of all the peaks, which is due to an increase in the lattice parameter with increasing Co composition. Lattice parameters determined from the fitting, increase linearly with increasing Co composition and can be expressed by the linear equation as in eqn (1) (Vegard's law), and is shown in Fig. 2(b).

1 *a*_Ni_1−*x*_Co_*x*_O_ = (1 − *x*) × *a*_NiO_ + *x* × *a*_CoO_

To determine directly the near neighbor Ni–O and Co–O bond lengths and distortions if any at the local level, EXAFS measurements were carried out at both the Ni and Co K-edges. The normalized EXAFS signals for the wave vector (*k*) values ranging from 3.43 Å^−1^ to 10.25 Å^−1^ (3.41 Å^−1^ to 10.22 Å^−1^) obtained at the Ni (Co) K-edges, have been Fourier transformed to obtain the Fourier components in real space coordinate (*R*) using hanning window function (with *dk* = 0.5). The structural parameters such as bond length and Debye–Waller factor (*σ*^2^) have been determined by fitting the EXAFS data. In the fitting, the co-ordination number has been fixed to six for RS crystal structure for first shell and for the second shell the co-ordination number has been fixed to twelve, where the ratios of Ni and Co have been considered according to their composition in the solid solution Ni_1−*x*_Co_*x*_O. The binaries NiO and CoO have been chosen as standard samples for determining the structure factor (*S*_o_^2^) and the same has been kept fixed for fitting the EXAFS data taken at Ni and Co edges.

**Fig. 3:**
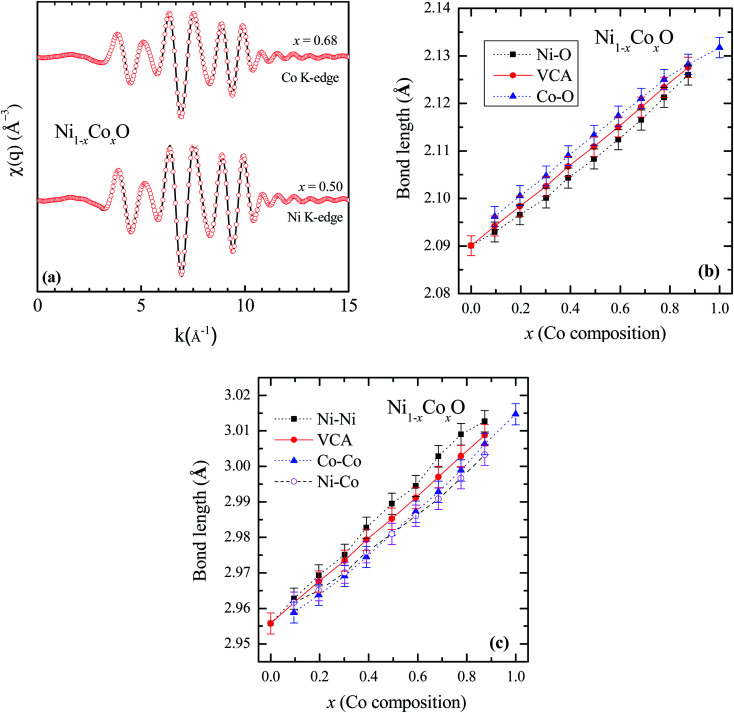
(a) EXAFS spectra [*χ*(*q*)] at Ni and Co K-edges for Co content of *x* = 0.5 and *x* = 0.68 in NiO. Solid lines represent experimental data and symbols are the fitted data. (b) First near neighbor (Ni–O/Co–O) bond length as a function of Co composition in Ni_1−*x*_Co_*x*_O. (c) Next near neighbor (Ni–Ni/Co–Co/Ni–Co) bond length. The solid line is for the data obtained from VCA and the symbols are fitted data in (b) and (c).

Based on the fitting of EXAFS data in real space, NN and NNN bond lengths have been determined for the Ni_1−*x*_Co_*x*_O ternary solid solutions. The back Fourier transformation of EXAFS signals for the values of R ranging from 1.2 Å to 4 Å are shown in Fig. 3(a) for two Co composition. This data includes the contribution from the first co-ordination shell (Ni–O, Co–O) and the second co-ordination shell (Ni–Co, Ni–Ni, Co–Co) for the EXAFS data at Ni and Co K-edges. The bond lengths of NN (Ni/Co–O) and NNN (Ni–Ni/Co–Co/Ni–Co) of the solid solution as a function of Co composition is shown in Fig. 3(b) and (c). The values of NN and NNN (Ni–Ni/Co–Co) bond lengths of NiO (CoO) are 2.090 ± 0.002 Å (2.127 ± 0.002 Å) and 2.956 ± 0.003 Å (3.015 ± 0.003 Å), respectively, which matches well with the reported values.^28,29^ The figure shows a linear variation of NN bond length as a function of Co composition throughout the series of Ni_1−*x*_Co_*x*_O, which closely follow the evaluated NN bond length variation as expected from the lattice parameter determined from XRD under the VCA.^30^ The value of bond length of NiO determined from XRD matches well with the reported values of bond length determined from XRD in literature.^31,32^ This observation is in sharp contrast to the observations reported in covalent tetrahedrally bonded (four-fold coordination), III–V ternaries like In_*x*_Ga_1−*x*_As^33^ and GaAs_*x*_P_1−*x*_,^34^ partially ionic tetrahedrally bonded, II–VI ternaries Zn_1−*x*_Be_*x*_Se,^35^ Cd_1−*x*_Mn_*x*_Te,^36^ ZnSe_*x*_Te_1−*x*_,^37^ Zn_1−*x*_Mn_*x*_S,^38^*etc.*, where the cation–anion near neighbor bond lengths are quite close to the parent binary compounds, and significantly deviate from the bond lengths evaluated from the lattice parameter values obtained from XRD. The NNN distances have also been addressed in some of the above reports, where a relatively smaller deviation from VCA has been observed. Similar deviation of the near neighbor bond lengths from VCA values, have also been reported for octahedrally bonded (six fold coordination), I–VII ionic ternaries like (K_1−*x*_Rb_*x*_)Br, and Rb(Br_1−*x*_I_*x*_).^39^ A review of similar experimental observations and their correlation with theoretical models have been summarized by Boyce *et al.*^40^ Interestingly, in Zn_1−*x*_Ni_*x*_O (upto *x* = 0.3), where the Zn and Ni atoms are octahedrally bonded with oxygen, it is observed that the Ni–O bond lengths show a behavior with “*x*”, which is consistent with VCA, whereas the Zn–O bond is relatively stiff and its length is close to the Zn–O bond in the parent ZnO system.^28^ Similarly, in Ni_1−*x*_Mg_*x*_O and Co_1−*x*_Mg_*x*_O, it is observed that the near neighbor distances Ni–O and Co–O follow closely the VCA derived values.^41^ They however report a small deviation from VCA for the Ni–Ni next neighbor distance in Ni_1−*x*_Mg_*x*_O.^42^ This behavior of cation–anion bonds in mixed crystals, having values close to their bond lengths, as in their parent binary compounds, is also referred to as relaxed behavior. This concept of a relaxed behavior was proposed by Bragg^43^ and Pauling,^44^ is based on the conservation of the covalent radii of the bond. The estimation of the change in the bond length of a cation–anion pair in a mixed crystal was addressed by Martins *et al.*^45^ where they estimated the change in elastic deformation energy, due to the presence of an anion/cation in a semiconductor crystal. They modeled the system by a valence force field (VFF), generalized to have the appropriate force constants for each kind of bond present in the mixed crystal. The appropriate force constants include the ones corresponding to the bond stretching and bond bending of each of the bonds in the mixed crystal. One of the important conclusions arrived at by Martins *et al.*, is that the cation–anion bonds in covalent mixed systems have a significant reduced relaxation (*i.e.*, compliant with VCA lattice) as compared to their ionic counterparts. L. Bellaiche *et al.* have also carried out a 1000 atom cluster calculation of the mixed crystal Ga_1−*x*_In_*x*_N and found that the system when evaluated with tetrahedral bonds (zinc blende or wurtzite structure) was on an average more relaxed as compared to the same system evaluated with an octahedral bonded RS structure.^46^ However, both the structures were significantly relaxed, and the individual Ga–N and In–N bond lengths were quite far from the VCA predicted values. Analyzing the above experimental reports and the corresponding theoretical explanation, we find that generally in mixed crystals, irrespective of whether the bond is ionic or covalent, whether the metal has a four-fold or a six-fold coordination, there is a tendency for the system to maintain their individual bond lengths of their parent binary compound.

Ni–O and Co–O bonds are however notable exceptions, which are unrelaxed, *i.e.*, they do not maintain their individual parent bond lengths, and follow the VCA derived bond length quite closely, and hence they are significantly more compliant with the average lattice of the mixed crystal. The variation of the NN bond lengths can be quantified by using the ratio: *S*/*S*^*v*^ ratio,^35^ where *S*^*v*^ is the slope of the bond length *vs.* “*x*” curve described with Vegard's law under VCA and *S* is the slope of the measured bond length *vs.* “*x*” curve. *S*/*S*^*v*^ is evaluated to be 18% in GaAs_*x*_P_1−*x*_ for both the Ga–As and Ga–P bonds. *S*/*S*^*v*^ in Zn_1−*x*_Be_*x*_Se have been evaluated to be 33% and 13%, for Zn–Se and Be–Se bonds respectively. These values evaluated for the Ni–O and Co–O bonds in Ni_1−*x*_Co_*x*_O are 99% and 96% respectively. As can be seen in Fig. 3(c), the second nearest neighbor bond lengths: namely, Ni–Ni, Ni–Co, and Co–Co, also nearly follow the bond lengths evaluated assuming VCA based structure. This important observation leads us to infer that unlike most of the conventional semiconductor ternaries, there is only a minimal distortion of the local structure about the Ni, Co and O atoms in Ni_1−*x*_Co_*x*_O system. Although the dependence of the bond lengths for covalent bonds with “*x*” is generally expected to be closer to VCA than ionic bonds,^45^ the Ni–O and Co–O bonds are not significantly more covalent than GaAs and InAs bonds, and thus covalency of the bonds alone cannot explain our results. Other factors like small difference between the Ni–O and Co–O bond lengths, strong p–d hybridization of the bonds, *etc.* also possibly contribute to this important observation.

In general, local distortion in a mixed crystal is expected to have important consequences on some of the properties in these mixed crystals. Prominent among them include the bowing in the optical gap of the ternaries^46^ scattering of carriers during transport,^47^ magnetic properties,^48^*etc.* The issue of optical gap in Ni_1−*x*_Co_*x*_O is discussed in detail, subsequently in the paper. We now briefly look at the antiferromagnetic to paramagnetic transition temperature (Néel's temperature *T*_N_) in this system.

**Fig. 4:**
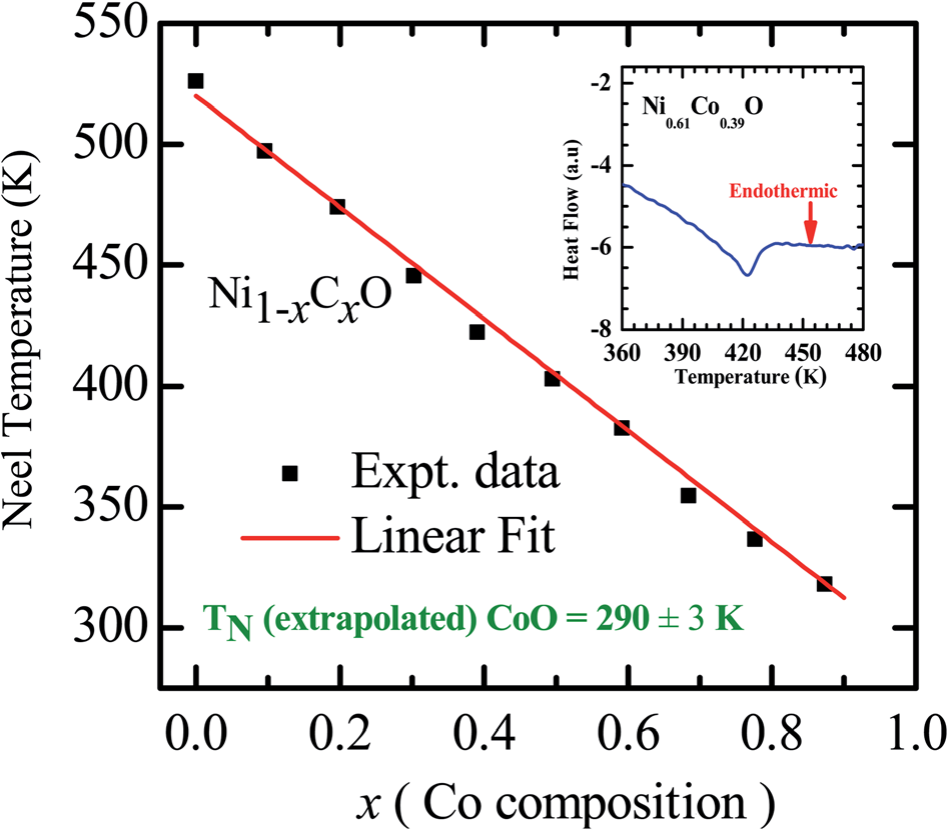
Néel temperature variation as a function of Co composition in Ni_1−*x*_Co_*x*_O solid solutions. Inset shows the DSC thermogram of Ni_0.61_Co_0.39_O.

As already mentioned, both NiO and CoO are type-II antiferromagnetic oxides, with a wide difference between their Néel temperatures. (NiO (*T*_N_) = 523 K; CoO (*T*_N_) = 291 K). The antiferromagnetic correlations in these systems are due to superexchange interactions, in which the two metal ions, (Ni/Co in this case) interact among themselves through the intermediate oxygen atom. The variation of the Néel temperature with substitution of Co in NiO is thus expected to alter the interaction between the metal ions and hence result in a variation of *T*_N_ with “*x*”. Fig. 4 shows the variation of *T*_N_ as a function of “*x*” in Ni_1−*x*_Co_*x*_O within ± 3 K. We see that there is a linear variation of *T*_N_ with “*x*” over the full range (*T*_N_(*x*) = 520 − 2.1 × *x*). This observation is in agreement with reported experiments on determination of *T*_N_ with “*x*” in Ni_1−*x*_Co_*x*_O and their estimation using molecular field approximation as reported by Bracconi *et al.*^49^.

We now look at the optical properties of this mixed system. The late transition metal 3d oxides are charge transfer insulators, whose band gap is due to the energy gap between the filled oxygen levels (which form the valence band) and the unfilled d levels (upper hubbard band) (which form the conduction band). Although the individual band gap of the NiO and CoO have been determined and analyzed by several authors,^1,18,50^ the variation of the gap with “*x*” in Ni_1−*x*_Co_*x*_O, which is of importance in band gap engineering, has not been addressed in detail over the complete composition range. The optical band gap of bulk Ni_1−*x*_Co_*x*_O ternary solid solutions has been determined by DRS. The diffuse reflectivity data have been converted into pseudo-absorbance spectra *F*(*R*_∞_) using Kubelka–Munk function according to the formula given below,^51^2
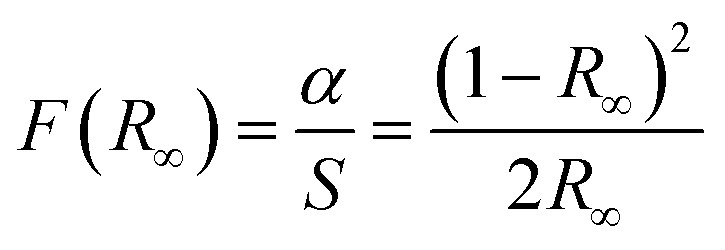
where *R*_∞_ is reflectance of an infinitely thick sample, *α* and *S* are absorption and scattering coefficients, respectively. Throughout the visible range of optical spectrum, the parameter *S* does not change, hence it can be considered as a constant. We analyse the absorbance spectra, *F*(*R*_∞_), using the Tauc's relationship, given by eqn (3), to find the gap of the solid solutions.^52^

**Fig. 5:**
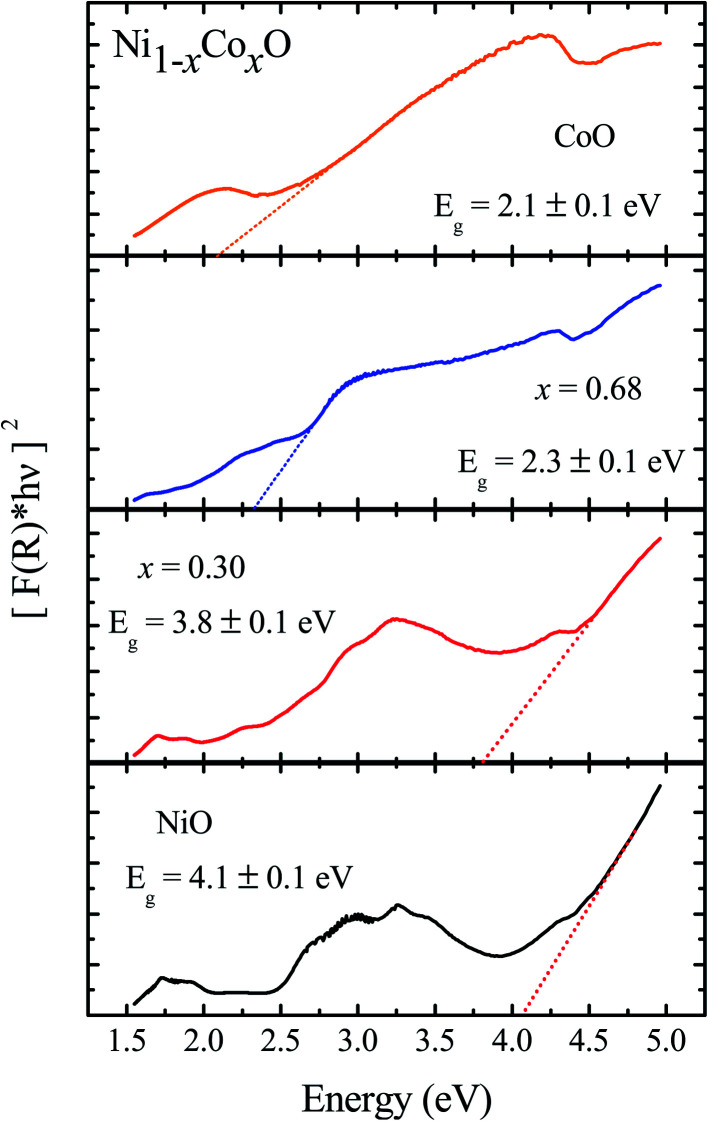
Diffuse reflectance spectra of Ni_1−*x*_Co_*x*_O for NiO, *x* = 0.3, 0.68 and CoO.

**Fig. 6:**
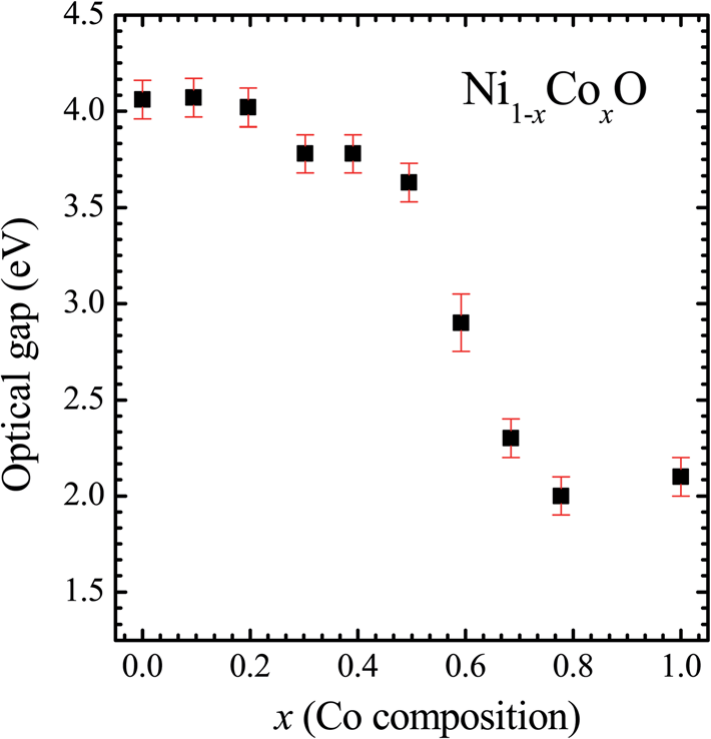
Optical gap for Ni_1−*x*_Co_*x*_O solid solutions as a function of Co content.

3 *F*(*R*_∞_)*hν* = *A*(*hν* − *E*_g_)^1/2^

where *A* is proportionality constant and *hν* is the incident photon energy. Graphically, the optical band gap, *E*_*g*_, has been obtained by extrapolating the slope of [*F*(*R*_∞_)*hν*]^2^*versus hν* to *α* = 0 as shown in Fig. 5 for a few Co composition. The values of optical gap obtained for the bulk ternary solid solution have been shown in Fig. 6. Various absorption peaks, below ∼4.0 eV, are also observed in the optical absorption spectrum of NiO shown in the Fig. 5. These absorption peaks are related to the bulk d–d excitations as noted by various authors in the optical absorption of NiO determined by different techniques. The absorption peaks observed at different energies, such as, ∼1.75 eV, ∼1.95 eV, ∼2.75 eV, ∼2.95, ∼3.25, and ∼3.52 eV are related to the transitions from ^3^A_2g_ (^3^F) triplet ground state to the triplet and singlet final states, ^1^E_g_ (^1^D),^3^T_1g_ (^3^F), ^1^T_2g_(^1^D), ^3^T_1g_ (^3^P), ^1^A_1g_ (^1^G) and ^1^T_1g_ (^1^G), respectively. The leading absorption edge at ∼4.01 eV in the optical absorption spectrum of NiO is related to the charge transfer from ligand (O) to metal (Ni) 3d states, and it is called the charge transfer gap or the optical gap.^1,27^ It is also attributed to the transition from the triplet ground state ^3^A_2g_ (^3^F) to singlet final state ^1^E_g_(^1^G). The leading absorption edge at ∼2.1 eV in the absorption spectrum of CoO is attributed to the excitation from quartet ground state, ^4^T_1g_ (^4^F), to quartet final state, ^4^A_2g_(^4^F).^1^The values of optical gap thus determined from DRS for the extreme members (NiO: 4.1 ± 0.1 eV and CoO: 2.1 ± 0.1 eV) of the series are in good agreement with the reported values.^1,50^ From Fig. 6 it is evident that the optical band gap neither changes linearly with Co composition nor has bowing as in most common ternary semiconductors. With increasing “*x*” the gap decreases slowly from 4.1 ± 0.1 eV to 3.6 ± 0.1 eV for the Co incorporation in NiO up to *x* = 0.5. After this, the optical gap suddenly decreases, and beyond *x* = 0.68 of Co incorporation, it remains almost constant at an optical gap value of ∼2.1 ± 0.1 eV. This behavior of the gap with composition is quite different from the ones that are reported in nearly all the ternary semiconductors, and hence it is important to investigate and understand the nature of this variation of gap with “*x*”. For this, the electronic structure (unoccupied states of the conduction band) of the samples has been investigated using XANES measurement at the O K-edge (1 s absorption). T. M. Roffi *et al.*,^53^ has reported the variation of optical gap for Co doped NiO thin films from 3.7 eV to 2.35 eV for the variation of *x* from 0 to 0.35. The change in optical gap is 1.35 eV which is larger than the change in the gap of 0.35 eV as observed in the present work for the same variation of *x*. The larger change in the energy gap may be due to the effect of stain developed in thin films. Variation of optical gap for Co doped NiO nano particles has been reported by P. Mallick,^54^ where the gap varies from 3.65 eV to 3.38 eV for the variation of *x* from 0 to 0.05. In both the studies, the variation of optical gap for the whole composition range, *i.e.*, *x* = 0 to 1, has been fitted by Vegard's law with bowing parameter of 3.5 eV and 5.28 eV, respectively, without providing values of energy gap in the whole composition range.

**Fig. 7:**
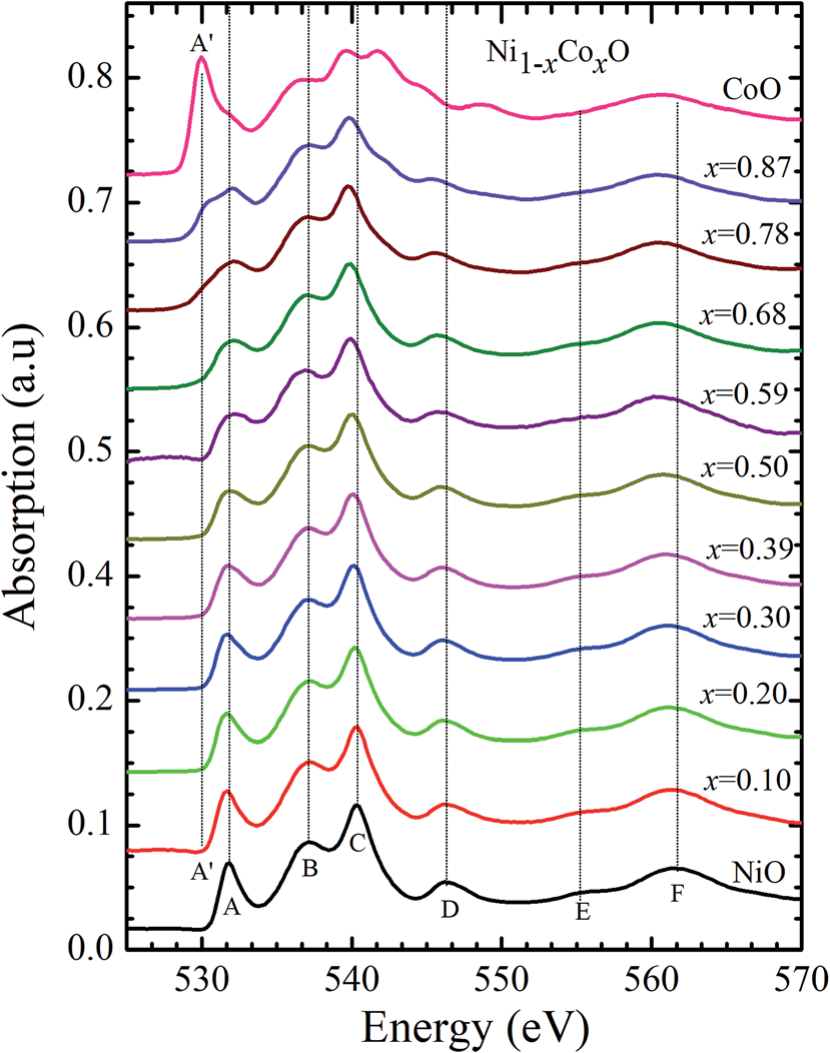
X-ray absorption spectra at O 1s edge for Ni_1−*x*_Co_*x*_O solid solutions as a function of Co content.

**Fig. 8:**
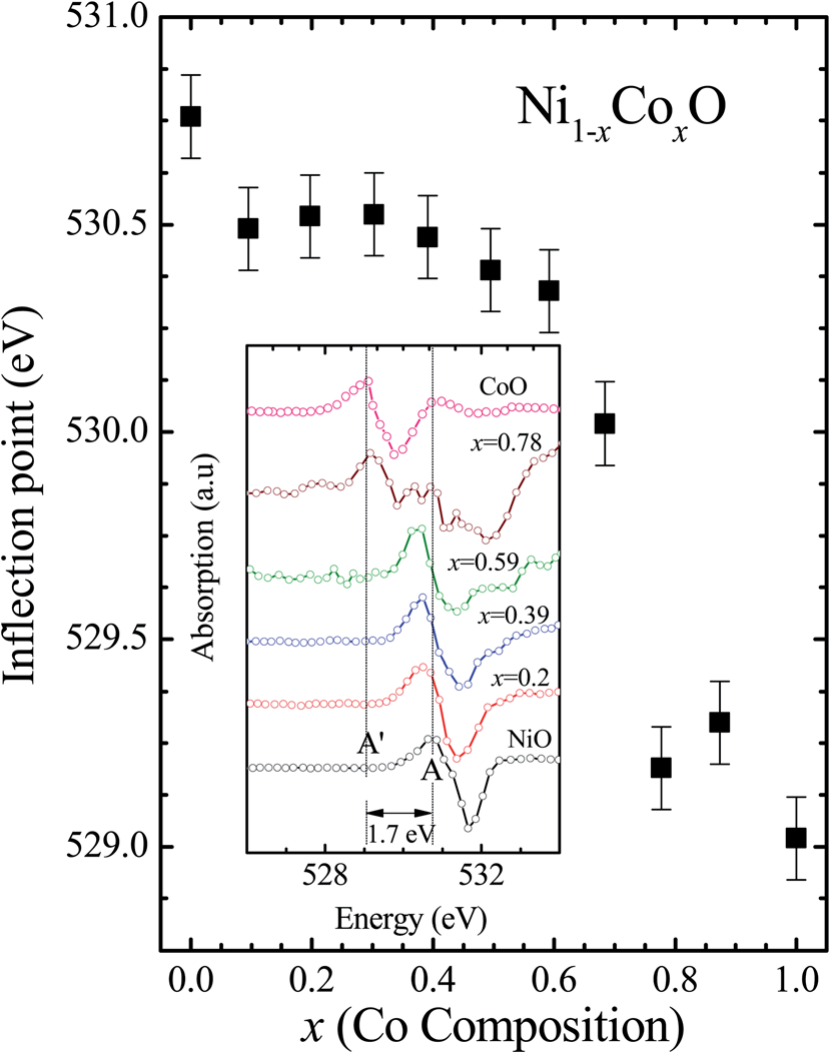
The inflection point of features A and A′ of Fig. (6) as a function of Co content in Ni_1−*x*_Co_*x*_O solid solution. Inset shows the 2^nd^ derivative of XAS spectra for features A and A′ as a function of Co content in the solid solution.

Kurmaev *et al.* have explicitly noted using experiments and calculations, that the O 1s absorption edge features in transition metal oxides, give a very good representation of the conduction band features as the O states are strongly hybridized with the metal 3d states and the O-2p XAS is only weakly affected by the O core hole.^18^Fig. 7 shows the XANES spectra at O K-edge for all the samples. We find that the onset of XAS signal in CoO is at about 2 eV lower, as compared to NiO. We next analyze all the features in the O 1s absorption spectra starting from *x* = 0 to *x* = 1. Typically, the spectra has six main features which are labeled by A, B, C, D, E and F, respectively. The feature A observed at ∼532 eV, in NiO (*x* = 0) represents the transition of O 1s electron to unoccupied states which is formed due to the hybridization of O 2p and narrow metal Ni 3d bands. The features B to F attributed to oxygen p character hybridized with Ni metal 4s and 4p states.^55^ According to multiple scattering theory, the feature B arises due to multiple scattering (MS) of photoelectrons between the absorber and the atoms located at the higher neighboring shells (long/medium range scattering), and the feature C arises due to MS within the first oxygen shell (short range scattering), as well as a feature at the threshold of unoccupied O 2p conduction band. The peaks D and E can be attributed to single scattering (SS) between the absorbing atoms and the atoms present at the higher neighboring shells, while the peak F arises due to single scattering within the nearest neighbor oxygen shell.^56^ The width of the feature A increases slowly with Co content in NiO up to *x* = 0.50, and for *x* = 0.59 and 0.68, the width significantly increases. Beyond *x* = 0.68, a new feature A′ (∼529 eV) becomes prominent, which is attributed to the transition of O 1s electron to unoccupied states formed due to the mixing of O 2p with Co 3d states. To determine the energy of onset of the conduction band the procedure used by Kurmaev *et al.*^18^ has been adopted. The lowest energy peak in the spectrum of the second derivative of the XAS spectrum for each sample has been determined to find the inflection point of the feature A or A′. These peak positions give the variation of conduction band edge, which is shown in Fig. 8.

It is clear from the above discussions and the figures, that the trends in the change of the conduction band features and the band gap with “*x*” are intimately related to the change in the electronic structure of Ni_1−*x*_Co_*x*_O with “*x*”. As the magnitudes of the change in the band gap with *x*, and the shift in the conduction band edge with *x* is very similar, it is fair to expect that the change in the band gap is primarily due to the energy shifts in the conduction band edge only. For low values of “*x*”, the bands are mainly dominated by the Ni 3d levels, with only a slight admixture of Co 3d states. With increasing “*x*”, *i.e.* with increasing Co concentration, the mixing of the Co 3d states with Ni 3d states increase. As the Co 3d states are more delocalized as compared to Ni 3d states (*U*_dd_ for Co in CoO is 4.7 eV, *U*_dd_ for Ni in NiO is 5.9 eV)^57^ the resultant bands broaden with increasing “*x*”. However, as the conduction band bottom is dominated by Ni e_g_ like states, there is no significant shift in the conduction band minima and hence in the O K-edge absorption data. This is given by the position A in the XAS data, which increases in its width with increase in “*x*”, but is nearly at the same position. As “*x*” crosses 0.59, *i.e.*, more than 59% of the metal sites are occupied with Co atoms, the Co *t*_*2g*_ states start to dominate and the feature A′ is seen in the XAS data. This feature develops into a proper peak with further increase in “*x*”, and its position approaches that of the Co t_2g_ state observed in the XAS data of CoO. Thus, a sharp transition between a NiO like conduction band minima to CoO like conduction band minima is observed in this ternary system at around *x* = 0.59. This therefore explains the reason behind the step like behavior in the band gap variation with “*x*”, which is indeed quite unique, when compared with standard semiconductor ternaries. Thus, the XAS data helps us to explain the variation of the optical band gap with “*x*” in Ni_1−*x*_Co_*x*_O ternary system. It may be however noted that the total change in the band gap with *x* is approximately 2 eV, which is 0.3 eV smaller than the shift of the conduction band edge with *x*, as determined from the oxygen edge XANES data. This can be attributed to small changes in the valence band maximum with Co concentration, *i.e.*, when *x* increases from 0 to 1. However, the principal cause for the observed trends in the variation of band gap with Co concentration remains the variations in the conduction band edge.

**Fig. 9:**
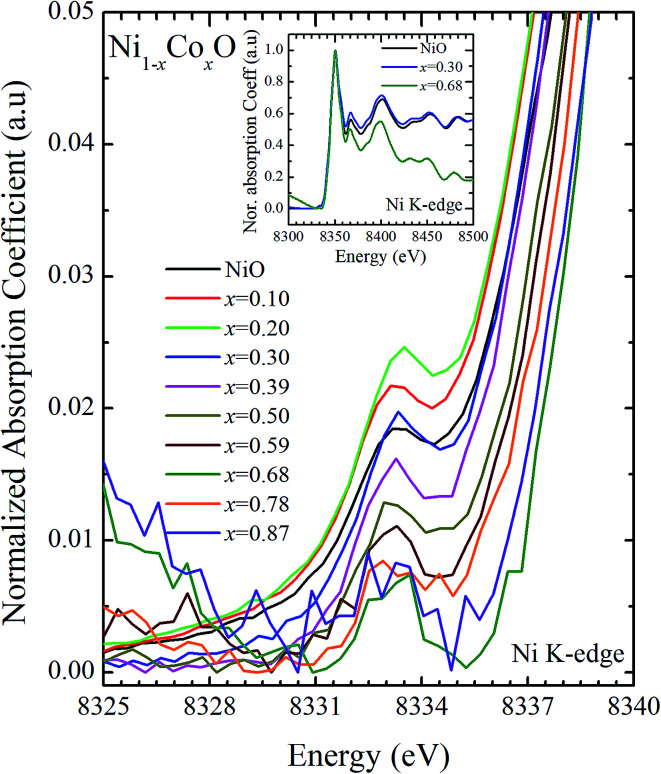
Pre-edge peak in XANES spectra measured at Ni-edge as a function of Co content in Ni_1−*x*_Co_*x*_O solid solution. Inset shows XANES spectra for NiO, *x* = 0.3 and *x* = 0.68 at Ni K-edge.

**Fig. 10:**
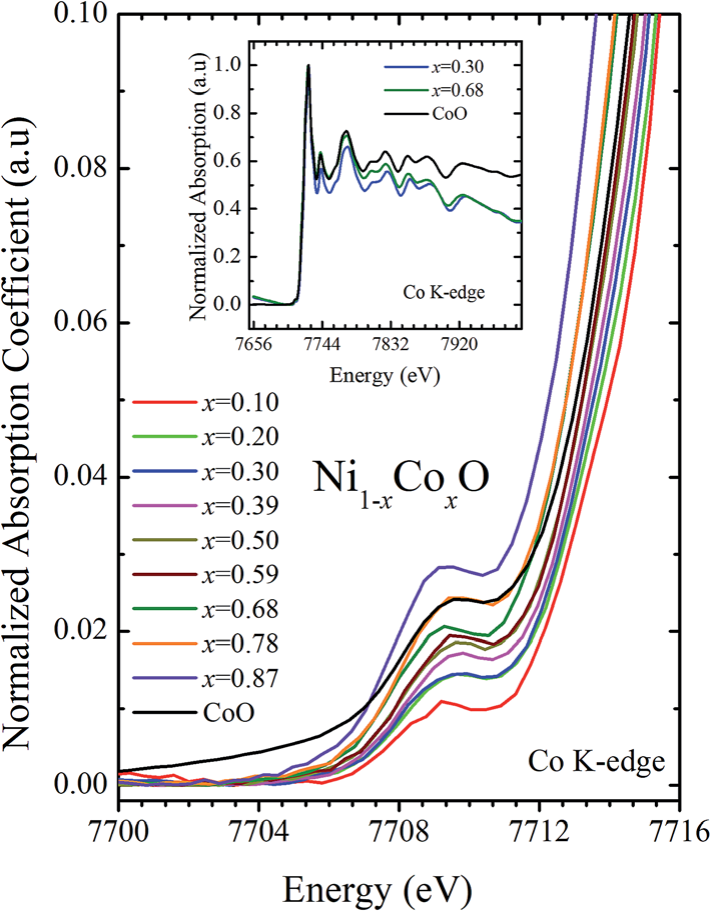
Pre-edge peak in XANES spectra measured at Co-edge as a function of Co content in Ni_1−*x*_Co_*x*_O solid solution. Inset shows XANES spectra for *x* = 0.30, 0.68 and CoO at Co K-edge.

**Fig. 11:**
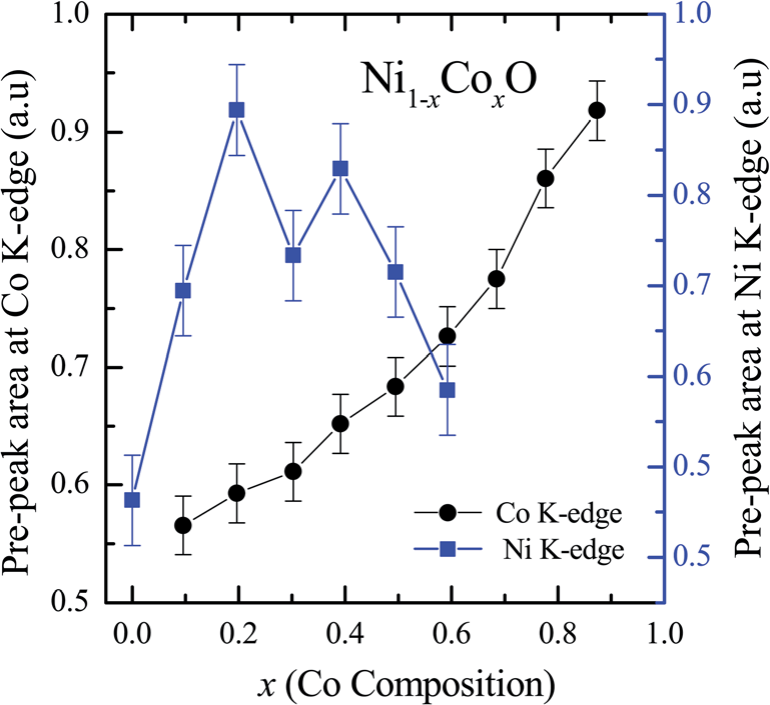
Pre-edge peak Intensity (area under the curve) in XANES spectra measured at Ni and Co K-edges as a function of Co content in Ni_1−*x*_Co_*x*_O solid solution.

XANES data at the Co and Ni K-edges have also been recorded. The data shows a distinct pre-edge feature. The pre-edge feature arises due to the transition from the 1s core electron of Ni and/or Co to the unfilled levels. Fig. 9 and 10 show the XANES data with the expanded view of the pre-edge data at the Co and Ni edges, respectively (insets show few representative XANES data). The intensity of the pre-edge feature depends upon various factors which include: the number of unfilled d levels into which the transition takes place (increase in unfilled d levels increases pre-edge intensity), the magnitude of hybridization of the 3d states with the O 2p states (increased O 2p – Ni(Co) 3d hybridization increases pre-edge intensity, because of larger transition probability of the dipole allowed transition from 1s to the p type unfilled states), and the variations in the Ni(Co)–O bond length in different samples (increase in bond length decreases O 2p – Ni(Co) 3d hybridization thereby decreasing the pre-edge intensity).^58^Fig. 11 shows the variation of the intensity (area) of the pre-edge feature of Co and Ni XANES with “*x*”. In all the cases, the data is normalized to the main edge feature of the XANES data for comparison. This normalization takes care of the trivial cause of change in the pre-edge intensity due to the concentration variation of the Ni/Co in the different samples. The Co pre-edge feature shows an increase in intensity with “*x*”. This can be explained from the fact that with increasing “*x*”, *i.e.*, with an increase in the fraction of Co, there is an increase in the number of empty d states. Thus, the intensity of the pre-edge feature at Co K edge increases with “*x*” due to the availability of increased number of empty d-states with increase in “*x*”. In fact, this factor dominates over the other opposing factor in which, with an increase in “*x*”, there is an increase in the average Ni(Co)–O bond length (it may be noted that EXAFS results clearly show that the Ni(Co)–O and Ni–Co bond lengths increase linearly with “*x*”, closely following the VCA prediction). Increased Ni(Co)–O bond length reduces the p–d overlap and hence a reduction in the percentage of O 2p contribution to the state. Reduced O 2p states reduces the possibility of transition of 1s electrons to the extended part of O 2p empty states, *i.e.*, reduces the s to p dipole allowed contribution to this transition. The Ni pre-edge peak intensity increases upto 20% of Co content in Ni_1−*x*_Co_*x*_O, although there is a depletion of Ni 3d empty states. This is attributed to the transition of 1s electrons to the extended part of empty O 2p-states as well as transition to empty Co 3d states because of smaller Ni/Co–O and Ni–Co bond lengths, respectively. The Ni pre-edge feature decreases in intensity with increase in the Co composition beyond 20%. This is primarily due to the fact that with increase in “*x*”, there is an increase in the Ni–O and Ni–Co bond lengths. The increase in Ni (Co)–O bond length reduces the p–d overlap and hence a reduction in the O 2p contribution to the bond. Increased availability of Co 3d states with increase in “*x*” does not significantly affect the Ni absorption intensity as these states, although mixed with Ni 3d states are physically centered on the Co atoms and hence have minimal effect on the Ni edge transition. The XAS data at L_2_-edge of Ni and Co have also been collected (figures not shown). The variation of peak intensity of L_1/2_ with respect to the normalized L_3/2_ edge is similar to the variation of peak intensity of pre-edge in XANES spectra measured at Ni and Co K-edges, respectively. The variation of peak intensity can be explained by similar arguments discussed above with the consideration of electron transition from metal p-states to empty O 2p metal (3d) hybridized state.

## Conclusion

The Ni_1−*x*_Co_*x*_O solid solutions have been characterized using XRD, EXAFS and XAS. The ternary solid solution is found in cubic RS phase, and the lattice parameter of the phase pure solid solutions is found to increase linearly with “*x*”, following the Vegard's law. The NN (Ni/Co–O) and NNN (Ni–Ni/Co–Co/Ni–Co) bond lengths determined from EXAFS, are found increasing linearly with “*x*”, in Ni_1−*x*_Co_*x*_O. The variation of these bond lengths nearly follow the variation of bond lengths evaluated from VCA, which is unlike most conventional ternary semiconductors, where the distortion in the local structure around the constituent atom is observed. The Néel temperatures of the solid solutions determined from DSC measurement is found to decrease linearly with “*x*”. The optical gap of the solid solutions determined from DRS measurement neither varies linearly with “*x*”, nor does the variation show any bowing as reported in several ternary semiconductors. With increase in “*x*”, upto *x* = 0.5, the gap decreases slowly from 4.1 eV to 3.7 eV. Beyond *x* = 0.5, the gap falls sharply to 2.1 eV, and beyond *x* = 0.68, it again remains almost constant at ∼2.1 eV. This variation of the optical gap has been explained by investigating the conduction band using XAS at O K-edge for different values of “*x*”. The conduction band edge is found almost at the same position for *x* upto 0.59 indicating the dominance of Ni 3d (e_g_) state, beyond *x* = 0.59, the edge moves to lower energies, due to the dominance of Co 3d (t_2g_) state. The trend in the variation of conduction band edge is similar to the variation of optical gap, which implies that the variations in the optical gap with “*x*” is strongly governed by the variations in the conduction band minima with “*x*”. The variation of pre-edge peak intensity with respect to normalized main peak in XANES spectra measured at Ni and Co K-edge, and the variation in the L_1/2_ peak intensity with respect to normalized L_2/3_ peak in XAS measured at L_2_ edge of Ni and Co are found to be similar, respectively. This trend in the variation of the peak intensity is found to be dependent on the unoccupied O 2p-metal (Ni/Co) 3d hybridized states and the bond lengths.

## Conflicts of interest

There are no conflicts of interest to declare.

## Supplementary Material
